# In Vitro Comparison of 2D-Cell Culture and 3D-Cell Sheets of Scleraxis-Programmed Bone Marrow Derived Mesenchymal Stem Cells to Primary Tendon Stem/Progenitor Cells for Tendon Repair

**DOI:** 10.3390/ijms19082272

**Published:** 2018-08-02

**Authors:** Chi-Fen Hsieh, Zexing Yan, Ricarda G. Schumann, Stefan Milz, Christian G. Pfeifer, Matthias Schieker, Denitsa Docheva

**Affiliations:** 1 Experimental Surgery and Regenerative Medicine, Department of Surgery, Ludwig-Maximilians-University (LMU), Nussbaumstr. 20, 80336 Munich, Germany; chifen.hsieh@med.uni-muenchen.de (C.-F.H.); matthias.schieker@med.uni-muenchen.de (M.S.); 2 Experimental Trauma Surgery, Department of Trauma Surgery, University Regensburg Medical Centre, Franz-Josef-Strauss-Allee 11, 93053 Regensburg, Germany; zexing.yan@ukr.de (Z.Y.); christian.pfeifer@ukr.de (C.G.P.); 3 Laboratory for Vitreoretinal Pathology, Department of Ophthalmology, LMU, Mathildenstraβe 8, 80336 Munich, Germany; ricarda.schumann@med.uni-muenchen.de; 4 Anatomische Anstalt, Lehrstuhl II, LMU, Pettenkoferstr. 11, 80336 Munich, Germany; stefan.milz@med.uni-muenchen.de; 5 Novartis Institutes for Biomedical Research (NIBR), Translational Medicine Musculoskeletal Disease, 4056 Basel, Switzerland; 6 Department of Medical Biology, Medical University-Plovdiv, 15A Vassil Aprilov Blvd., 4002 Plovdiv, Bulgaria

**Keywords:** mesenchymal stem cells, Scleraxis, tendon stem/progenitor cells, cell sheets, tendon repair

## Abstract

The poor and slow healing capacity of tendons requires novel strategies to speed up the tendon repair process. Hence, new and promising developments in tendon tissue engineering have become increasingly relevant. Previously, we have established a tendon progenitor cell line via ectopic expression of the tendon-related basic helix-loop-helix (bHLH) transcription factor Scleraxis (Scx) in human bone marrow mesenchymal stem cells (hMSC-Scx). The aim of this study was to directly compare the characteristics of hMSC-Scx cells to that of primary human tendon stem/progenitors cells (hTSPCs) via assessment of self-renewal and multipotency, gene marker expression profiling, in vitro wound healing assay and three-dimensional cell sheet formation. As expected, hTSPCs were more naive than hMSC-Scx cells because of higher clonogenicity, trilineage differentiation potential, and expression of stem cell markers, as well as higher mRNA levels of several gene factors associated with early tendon development. Interestingly, with regards to wound healing, both cell types demonstrate a comparable speed of scratch closure, as well as migratory velocity and distance in various migration experiments. In the three-dimensional cell sheet model, hMSC-Scx cells and hTSPCs form compact tendinous sheets as histological staining, and transmission electron microscopy shows spindle-shaped cells and collagen type I fibrils with similar average diameter size and distribution. Taken together, hTSPCs exceed hMSC-Scx cells in several characteristics, namely clonogenicity, multipotentiality, gene expression profile and rates of tendon-like sheet formation, whilst in three-dimensional cell sheets, both cell types have comparable in vitro healing potential and collagenous composition of their three-dimensional cell sheets, making both cell types a suitable cell source for tendon tissue engineering and healing.

## 1. Introduction

Tendons are dense connective tissues connecting muscle to bone, thereby transferring forces onto joints that are essential for locomotion. Tendon injuries may result in joint dysfunction because they often restore biomechanical and structural properties inadequately, produce a scar tissue matrix with inferior quality and are prone to be reinjured. Tendon injuries are a frequent medical problem and are mainly associated with overuse activity and age-related degeneration. Degenerative tendinopathy is considered to be one of the leading causes for tendon rupture, and it can also result in a slow and ineffective healing response and increased risk of re-rupture [1]. Tendon healing follows the classical wound closure process that is comprised of three conjunct steps, namely inflammation, proliferation and remodeling, and specifically for tendon tissues, the whole process can extend over one year [2,3]. For this repair cascade, the activation, migration, proliferation and differentiation of endogenous stem/progenitor cells are all very critical. Due to their poor repair capacity, novel strategies to speed up the tendon repair process and to maximize the functional outcome are required. One promising approach is tissue engineering based on the reparative potential of autologous adult stem cells delivered to the site of tissue damage alone or in combination with biocompatible scaffolds or gels [2,3]. With respect to the clinical need and satisfactory results, further research into the stage of pre-clinical development and optimization is required [4]. Two critical factors need to be considered, firstly selecting the most appropriate cell type, and secondly clarifying the optimal mode of cell delivery depending upon the type of disease and stage. The main endogenous tendon cell types are terminally differentiated tenocytes that are deeply embedded in between the collagen fibers and tendon stem/progenitor cells (TSPCs) that can be found in the intrafascicular matrix, epitenon and endotenon sheets, as well as in close proximity to vessels [2]. TSPCs are an attractive cell type for cell-based therapies, as they play a critical role in the tendon healing process and have the innate propensity to differentiate into tenocytes [5]. We have established the isolation and cultivation of TSPCs from human Achilles tendons (hTSPC) as well as characterized TSPCs in detail using in vitro and in vivo models. The hTSPCs exhibit typical adult stem cell features: positivity for stem cell markers (CD73, CD90, CD105, CD146, Nestin and STRO-1), trilineage differentiation potential and expression of high levels of tendon-related genes, such as collagen type I (COL1) and collagen type III, and the proteoglycans biglycan (BGN), cartilage oligomeric matrix protein (COMP), decorin (DCN), fibromodulin (FMOD), lumican (LUM), tenascin C (TNC), and versican [6,7]. Moreover, using a full-size injury model in rat Achilles tendon, we demonstrated that hTSPCs implanted as pellets or together with carriers lead to enhanced tissue repair [8,9]. Despite their multiple benefits, the major disadvantages of autologous hTSPCs are the comorbidity and the time required for their isolation and expansion prior to use. Therefore, another strategy to gain similar cells is a direct tenogenic programming of well-known and easily obtainable adult stem cells, such as bone marrow-derived mesenchymal stem cells (BM-MSCs) [10,11,12,13]. In 2012, we lentivirally transduced immortalized BM-MSCs with the cDNA of the key tendon transcription factor Scleraxis (hMSC-Scx) and revealed that SCX overexpression in this cell type results in reduction of self-renewability, inhibition of osteogenic and chondrogenic commitment, and upregulation of various tendon-related genes [11]. Furthermore, ruptured rat Achilles tendons treated with hMSC-Scx cells showed advanced cellular organization and matrix maturation, and negligible ectopic calcification [14].

Based on these results, we proposed the direct tenogenic programming of MSCs as an auspicious strategy to gain cells that are of therapeutic cells for tendon tissue repair. However, an important question that has to be addressed prior to clinical application is how similar the genetically modified MSCs are to native tendon-derived TSCPs. Hence, in this study, we directly compared primary hTSPCs to the immortalized single cell-derived cell line hMSC-Scx by analyzing their expression profiles (tendon-, mesenchymal lineage-related and collagen cross-linking genes), migration patterns, and ability to self-assemble tendon-like three dimensional (3D) cell sheets [3]. Cell sheet formation is based on a natural establishment of cell–cell and cell–matrix connections during cell culture with subsequent folding into a 3D tube-like shape [15,16]. Cell sheet technology resembles a process very similar to native tissue morphogenesis during embryo development; it is scaffold-free, as it not only eliminates the need of natural and synthetic carriers, but also offers a model to study in vitro tenogenic differentiation. 

## 2. Results

### 2.1. Comparison of Stem Cell Characteristic

**Figure 1:**
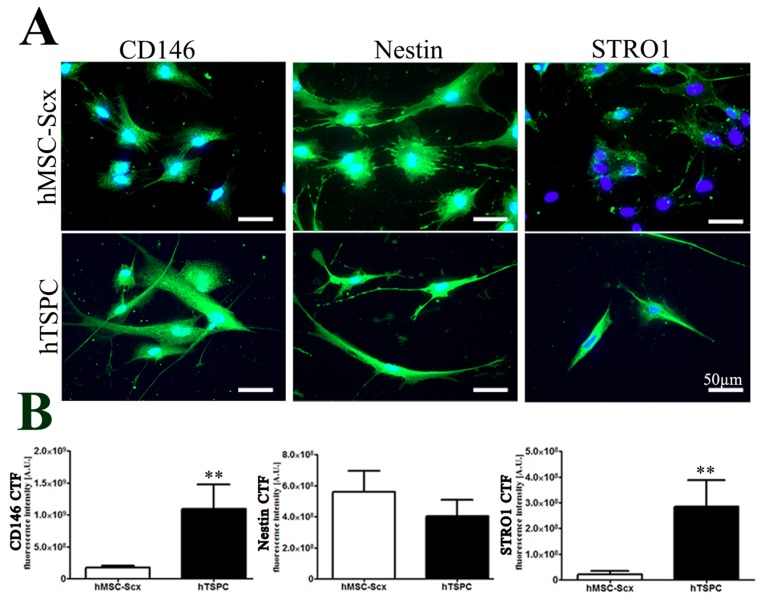
Expression of stem cell markers in hMSC-Scx cells and hTSPCs. (**A**) Representative fluorescent images of CD146, Nestin and STRO1 (in green color) and nuclear counterstain DAPI (in blue). (**B**) Digital quantification of CD146, Nestin and STRO1 fluorescent intensity levels. Bar charts represent mean ± standard deviation; ** *p* < 0.01, *n* = 3 (three independent experiments per cell type).

**Figure 2:**
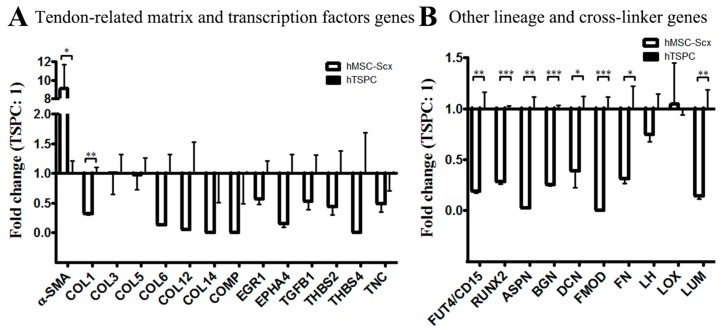
Gene expression profiling of 2D hMSC-Scx cell and hTSPC cultures. (**A**) QPCR for tendon related genes. (**B**) QPCR for other lineage-related and cross-linking genes. Statistical significance: * *p* < 0.05, ** *p* < 0.005 and *** *p* < 0.001, *n* = 3 (three independent experiments per cell type). Only genes with significant change in expression are plotted. For full gene lists and gene names, refer to Table 1 and Table 2.

First, morphological differences between hMSC-Scx cells and hTSPCs were monitored by phase-contrast microscopy (Figure S1A), and then cell territories were measured showing that hMSC-Scx cells exhibited bi- or tri-polar cell shapes and occupied approximately a 50% larger cell area than hTSPCs (Figure S1B). Self-renewal potential of both cell types was investigated by two different assays. Short-term WST-1 (48 h) (Figure S1C) and long-term DNA quantification based assays (16 days) (Figure S1D) demonstrated that the proliferation rate of hMSC-Scx cells is similar to that of hTSPCs. However, an evaluation of the clonogenic potential, by estimating the colony forming unit (CFU) efficiency, of both cell types, revealed that hMSC-Scx cells formed significantly less colonies than hTSPCs (Figure S1E). Next, hMSC-Scx cells and hTSPCs were subjected to adipogenic, chondrogenic, and osteogenic differentiation protocols, revealing that hMSC-Scx cells were able to commit into adipocytes, whilst hTSPCs successfully differentiated into the three lineages. This conclusion was validated for each lineage by corresponding staining and quantification, as well as by semi-quantitative PCR analyses for the expression of typical gene markers (Figures S2 and S3). Lastly, we performed immunocytochemistry and digital fluorescence intensity quantification for three well-accepted stem cell markers—CD146, Nestin and STRO1 (Figure 1A,B). The markers were expressed in both cell types; however, lower fluorescence intensity was detected for CD146 and STRO1 in hMSC-Scx cells (Figure 1B). Quantitative PCR was implemented to evaluate the expression levels of tendon-related matrix genes, transcription factors, and other lineage-related and collagen cross-linking genes in hMSC-Scx cells and hTSPCs (Figure 2, and Table 1 and Table 2). Significant differences were found in *COL1*, embryonic stem cell marker *FUT4/CD15*, osteogenic master transcription factor Runt-related transcription factor 2 (*RUNX2*) and several collagen cross-linking genes (Figure 2A,B). Therefore, the above data indicated that hTSPCs are more naïve and uncommitted than hMSC-Scx cells.

### 2.2. In Vitro Wound Repair Potential of the HMSC-Scx Cells Versus HTSPCs

**Figure 3:**
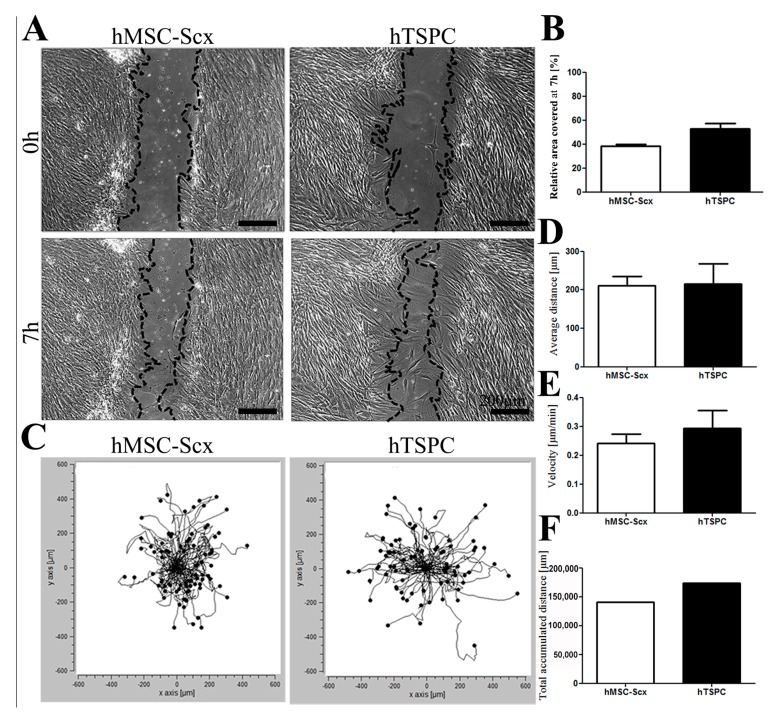
HMSC-Scx and hTSPC migration potential. (**A**) Representative images of wound closure at the beginning and the end of the experiment. The cellular fronts are outlined with black lines. (**B**) Calculation of area covered by cells in % to the initial wound area. Nine scratches per cell type were analyzed (*n* = 9). (**C**) Plots showing the forward migration index, where each black line is an individual cell track. (**D**–**F**) Quantification of the average and total accumulated distances and the cell velocity (*n* = 320 tracks per cell type).

For mimicking the wound healing process, we performed time-lapse imaging of in vitro scratch closure as well as random cell migration assays (Figure 3). The scratch assays revealed comparable closure rates for both cell types (Figure 3A,B). Plots of the forward migration index (FMI) and calculation of average and accumulated migration distances, as well as the migratory velocity, also demonstrated similar migratory potential of the MSC-Scx cells to hTSPCs (Figure 3C–F). In summary, our results showed that both cell types had very similar migratory behavior, which is an important feature during wound healing.

### 2.3. Qualitative and Quantitate Examination of Three-Dimensional hMSC-Scx and hTSPC Sheets

**Figure 4:**
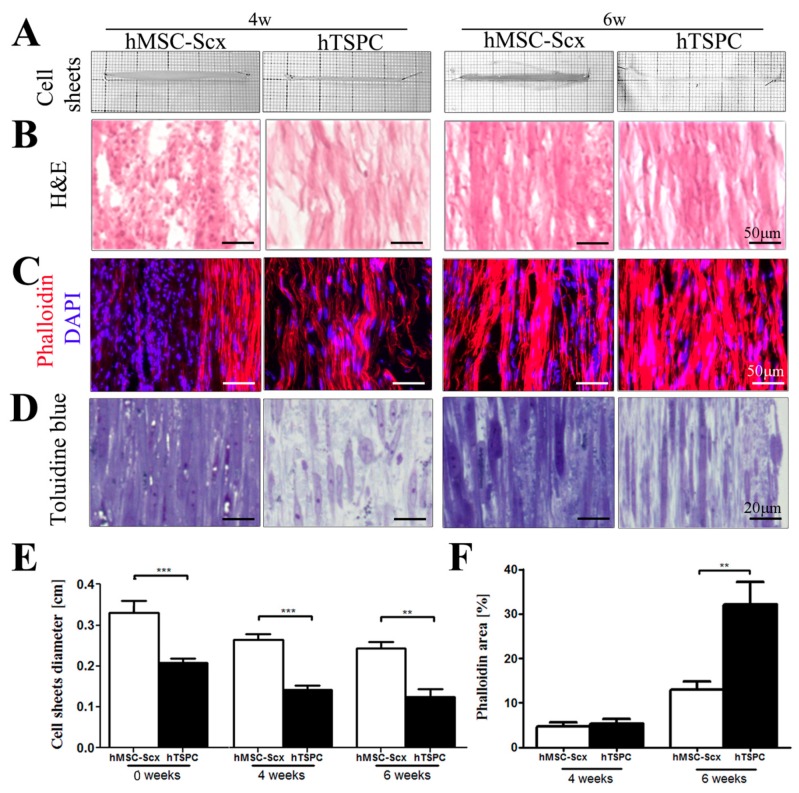
Cell sheet morphology and size. Representative gross morphological (**A**) and H&E (**B**), phalloidin (**C**) and toluidine blue (**D**) images taken at 4w or 6w after sheet assembly. Quantification of average sheet diameters of hMSC-Scx cells and hTSPCs at 0 (day of sheet assembly), 4w and 6w. (**E**) and phalloidin-positive area (**F**) at both examined time points. 4w, 4 weeks; 6w, 6 weeks.

**Figure 5:**
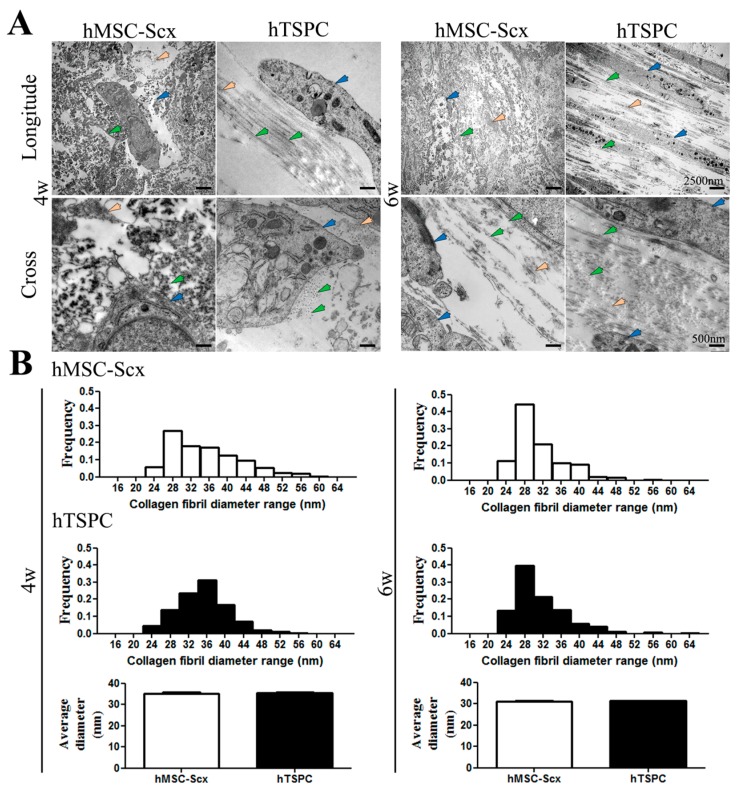
TEM and collagen fibril diameter analyses. (**A**) Representative TEM images from cross and longitudinal sections of hMSC-Scx and hTSPC sheets at 4w or 6w of maturation (cells indicated with blue arrow heads, collagen-like fibers indicated with green arrow heads and other unknown fibers indicated with orange arrow heads). (**B**) Frequency distribution of various collagen fibril diameters and average diameters of both types of cell sheets at 4w or 6w. 4w, 4 weeks; 6w, 6 weeks.

**Figure 6:**
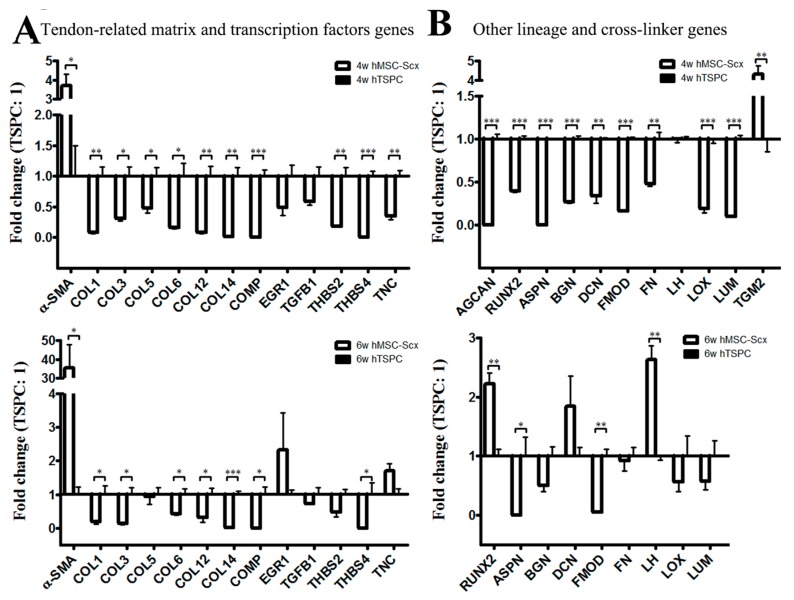
Gene expression profiling of 3D hMSC-Scx and hTSPC sheets. (**A**) QPCR for tendon-related genes. (**B**) QPCR analysis for other lineage-related and collagen cross-linking genes. Statistical significance: * *p* < 0.05, ** *p* < 0.005 and *** *p* < 0.001, *n* = 3 (three independent experiments per cell type and per time point). Only genes with significant change in expression are plotted. For full gene lists and gene names, refer to Table 1 and Table 2. 4w, 4 weeks; 6w, 6 weeks.

Gross appearance, cellular and matrix organization and composition of hMSC-Scx and hTSPC sheets were evaluated by cell sheet imaging (Figure 4A), H&E (Figure 4B), Phalloidin for F-actin (Figure 4C) and Toluidine blue (Figure 4D) staining at 4 and 6 weeks after cell sheet folding. Furthermore, cell sheet diameters and Phallodin-positive regions were measured (Figure 4E,F) at both time points. In general, hMSC-Scx cells formed significantly larger sheets with a matrix that was more amorphous and abundant for proteoglycans and glycosaminoglycans. In contrast, hTSPC sheets were very compact and their matrix appeared more fibrous and aligned. For both cell types, a maturation of the cell sheets from 4 to 6 weeks was observed, which was judged by a slight reduction in sheet size, higher matrix order and cellular alignment. There was an improvement in cell shape and elongation according to the Phalloidin staining and quantification of F-actin organization, representing cell shape and cell elongation were improved between 4 and 6 weeks for both cell types (Figure 4C,F). Transmission electron microscopy (TEM) images of longitudinal and cross sections confirmed the presence of a more fibrous matrix and elongated parallel cells in hTSPC sheets (Figure 5A). However, quantitative analyses of collagen fibril diameters (Figure 5B) showed no significantly different fibril size between hMSC-Scx cells and hTSPCs for both examined time points. Altogether, in comparison to hMSC-Scx cells, hTSPCs formed denser and more fibrous sheets enriched in aligned spindle-shaped cells, but the lateral growth of the collagen fibrils was comparable between the two cell types. Finally, we carried out quantitative PCR for 48 different genes with mRNA from the hMSC-Scx and hTSPC sheets collected at 4 or 6 weeks (Figure 6 and Table 1 and Table 2). In general, hMSC-Scx sheets showed lower expression levels of multiple genes; however, the fold-difference became smaller from 4 to 6 weeks, indicating hMSC-Scx sheet maturation. Only seven genes, namely alpha smooth muscle actin (*α-SMA*), Early growth response 1 *ERG1*, *TNC*, transglutaminase 2 (*TGM2*), *RUNX2*, *DCN* and lysyl hydroxylase (*LH)*, were up-regulated in hMSC-Scx versus hTSPC sheets. Taken together, our detailed analysis demonstrated that hTSPCs formed 3D self-assembled cell sheets with more mature and organized morphology in a faster manner than hMSC-Scx cell line.

## 3. Discussion

In recent years, vast research on cell-based tissue engineering has not only suggested, but also provided evidence for a promising forward concept for musculoskeletal tissue repair. In this approach, finding the most appropriate cell type and mode of application is critical to enable desired outcomes. Previously, we have established a hMSC-Scx cell line [11]. SCX is a bHLH transcription factor important for tendon development [17], and upon our investigation, we concluded that hMSC-Scx cells were enforced into the tendon lineage. We have also obtained primary hTSPCs from young and healthy non-ruptured Achilles tendons [6], and here, we directly compared how close hMSC-Scx cells are to hTSPCs. The data presented in this study clearly demonstrated that the hMSC-Scx cells and hTSPCs have comparable proliferation capacity, migratory behavior and deposition of collagen fibers in their 3D cell sheets. However, the results from CFU assay, trilineage differentiation and stem cell marker expression levels indicated that hTSPCs are more naive than hMSC-Scx cells. Moreover, hTSPCs form compact, denser and more mature 3D cell sheets in a shorter time period.

Several reviews have discussed that stem cells are in a “standby”-like mode expressing low levels of various lineage-associated transcription factors, and hence, ready to go upon appropriate stimulation in multiple alternative directions, while progenitor cells are more restricted in their fate decisions [18,19,20]. Indeed, our qPCR data shows in 2D and 3D cultures that, in hMSC-Scx cells, most of the screened genes are expressed to a lower extent. There are several possible explanations: firstly, in contrast to hMSC-Scx cells, the primary cells have parallel expression of other tendon-related transcription factors, such as EGR-2, EYA2, MKX, which can drive matrix gene expression (collagens), and secondly, the genetic modifications needed to generate this cell line might have an indirect influence.

Stem cells also have an ability to migrate throughout embryonic development and are found to reside in virtually any tissue. At different stages of embryogenesis, stem cells migrate long distances to new locations and start to specialize under exposure to tightly regulated biochemical and biophysical stimuli. Following tissue and organ formation, resident adult stems cells remain one of the few cell types that retain the capacity to self-renew, migrate and differentiate when activated, enabling them to maintain tissue homoeostasis and to mediate repair and regeneration [21]. Furthermore, there is growing evidence, claiming that the efficiency of stem cell homing can help to determine the degree of tissue regeneration that can be achieved [22]. Interestingly, our results from scratch assays and time-lapse monitoring of random migration showed no significant differences between hMSC-Scx cell and hTSPC migratory potential. One reason for this might be that hTSPCs are not a homogenous population, as they have not been obtained by cell sorting. Therefore, further research on purified stem cells and subsets of progenitors will be necessary for gaining a better understanding of the underlying molecular and cellular responses involved in homing, and can also lead to optimized therapeutic applications by identifying dominant regulatory mechanisms that can improve the integration of injected cells or implanted tissue engineered constructs. 

Cell sheet technologies based on a scaffold-free, self-assembly process are a powerful way to fabricate natural matrix-rich patches for repair of tendon defects. Furthermore, cell sheet engineering for tendon repair allows creation of multi-layered cellular architectures within their own microenvironment and establishes autocrine and paracrine signaling to the nearby tissues or interfaces at the point of implantation. Our investigation provides very novel and interesting findings that the hTSPC sheets are superior and form quicker than hMSC-Scx cell sheets. The hMSC-Scx sheets were significantly larger, contained more proteoglycan- and glycosaminoglycan-rich matrices and had slower maturation rates. Furthermore, they expressed higher mRNA levels of the transcription factors *EGR-1* and *RUNX2*, as well as regulatory genes of collagen fibrilogenesis, such as *TGM2*, *LH* and *DCN*. The up-regulation of *EGR-1*, known to act on collagen gene expression and to be involved in tendon maturation [10], might explain why, in terms of collagen fibril properties, both types of cell sheets were comparable. Still, future studies should aim to identify ways that can speed up the tendon-like sheet formation of not only hMSC-Scx cells but also other cell sources, such as primary bone marrow- or adipose-derived MSCs. One possibility investigation is the application of growth factors alone or in combination with mechanical stimulation. For example, in vivo delivery of recombinant bone morphogenetic protein-12 (rBMP-12) microspheres and autologous adipose-derived MSC sheets accelerated the proliferative stage of tendon healing whilst suppressing the adverse inflammatory reactions [23]. Others have found that stretching of 3D engineered tendons augmented the collagen stabilization and mechanical properties of the constructs [24,25].

HMSC-Scx cells have ectopic levels of this transcription factor that is known to activate the *COL1* gene in mesenchymal cells and to be part of a transcriptional network involved in the differentiation of such cells into the tendon and enthesis lineages. *Scx* gene ablation in *Mus musculus* results in profound defects in the development of intermuscular and force-transmitting tendons, which are concomitant with changes in collagen and other tendon-related gene expression [26,27,28]. Scx and the pro-chondrogenic transcription factor, Sox9, are detected in a double-positive subpopulation of embryonic progenitors that can alternatively enter in tendon/ligament or cartilage commitment during the early stages of musculoskeletal development [29]. Using a loss-of-function approach, Sugimoto et al., elegantly demonstrated that Scx+/Sox9+ progenitors functionally contribute to the establishment of the junction between hyaline cartilage and tendon/ligament tissues [30]. Scx is up-regulated during tendon formation, for example, in Achilles and patella tendons and the cruciate ligaments of the knee joint, whereas its expression is down-regulated at the site of ongoing chondrogenesis. Sox9 expression in the cartilaginous primordia endures during chondrogenic development, and then it is followed by Runx2 that orchestrates the osteoblast differentiation program. These lines of evidence suggest that the coordinated expression of Scx, Sox9 and Runx2 are tightly regulated in order to determine and sustain the cell populations of tenocytes, chondrocytes and osteocytes [31,32]. Hence, deciphering the exact crosstalk between Scx- and Sox9/Runx2-dependent developmental axes can be very helpful for understanding how the three lineages specify and operate. 

An important reason to consider for the detected discrepancy between hTSPC and hMSC-Scx sheet formation is that the latter are genetically engineered by lentiviral delivery of SCX and TERT cDNAs [11]. The genomic integration of the lentiviral construct can cause unknown changes in the cells. Therefore, it would be important in follow up research to investigate if MSCs from various tissues sources can be tenogenically committed by Scx overexpression in a non-viral, transient manner. Several papers have already discussed non-viral based gene transfer strategies for enhancing musculoskeletal engineering and healing [33,34,35]. One drawback of this study, namely donor matching in gender and age, should also be addressed in future investigations.

From a translational point of view both strategies—autologous hTSPCs or allogenic hMSC-Scx cell transplantation are possible, but the selection depends on the patient case. Our study demonstrated that autologous hTSPCs have multiple advantages; however, the main drawbacks for their use are patient morbidity and cell expansion time, as well as age-related reduction in cell quality, as reported by Kohler et al. 2013 [6]. Still, autologous tenocyte (obtained via patellar tendon needle biopsy) injections have been carried out to treat degenerative tendons and encouraging results were reported [36]. The hMSC-Scx cells might become an attractive source, especially for aged individuals where allogenic hMSCs isolated from bone marrow or adipose tissue of young donors can be programmed into tendon progenitors. Despite their several inferior features, hMSC-Scx cells were comparable to hTSPCs with regards to in vitro healing potential and collagenous composition of the cell sheets, as well as they aided in advanced cellular organization, matrix maturation, and negligible ectopic calcification when implanted in large defects in rat Achilles tendons [14]. However, to achieve this purpose, is very critical to establish a transient viral or a non-viral approach to circumvent the safety concerns related to genetic modification of donor-derived human cells.

Taken together, our study provides novel and exciting findings for further in vitro and in vivo research on Scx-steered tendon cell sheet engineering for promising clinical applications.

## 4. Materials and Methods

### 4.1. Cell Culture

In this study, two cell types previously characterized, namely the immortalized single cell-derived hMSC-Scx cell line from Alberton et al. 2011 [11] and primary hTSPCs from Kohler et al. 2013 [6], were used. For the generation of hMSC-Scx cell line (single cell-derived clone number 20), ileac crest bone marrow-derived hMSCs (donor: Lot-Nr. 4F0760, female 25 years old) were purchased from Cambrex (East Rutherford, NJ, USA). The company obtained the cells by density gradient centrifugation and controlled their quality by expression of positive CD29, CD44, CD105 and CD166 (>96%) and negative CD14, CD34 and CD45 (<5%) surface markers, as well as three lineage differentiation capacity. Primary hTSPCs were generated from three human non-injured Achilles tendons (males with a mean age of 28 ± 5 years). The isolation of hTSPCs was approved by the Ethics Commission of the Clinic of Ludwig-Maximilians-University (21 May 2008) and registered under the Ethical Project Number 166-08. Written informed consent was obtained from all participants. Briefly, cells were isolated via collagenase II digestion and filtration (100 µm pore size) and fully characterized by FACS for CD marker expression, gene expression profile, self-renewal and trilineage differentiated assays in Kohler et al. 2013 [6]. Based on the published in [6] growth characteristics, donor Y3-TSPCs (male, 33 years old) were implemented for representative imaging. Throughout the study, cells were maintained in the original media used for their generation, namely, for hMSC-Scx cells, Alpha minimum essential medium Gluta-MAX culture media (Gibco, Darmstadt, Hessen, Germany) supplemented with 10% fetal bovine serum (FBS, Sigma-Aldrich, Steinheim, Nordrhein-Westfalen, Germany) and for hTSPCs, DMEM/Ham’s F-12 (1:1 mixture) medium supplemented with stable glutamine (365.3 mg/L), 1 × MEM amino acids (all from Biochrom, Berlin, Germany), 10% FBS and 1% L-ascorbic acid-2-phosphate (both from Sigma-Aldrich, Steinheim, Germany) in order to avoid unknown effects on the cells upon medium switch. Prior use, hMSC-Scx cells and hTSPCs were cryopreserved in their corresponding media enriched with 10% DMSO and 20% FBS and stored in liquid nitrogen. Cell expansion was carried out on polystyrene dishes in a humidified incubator (37 °C and 5% CO_2_), and cells were used for experiments at 3 consecutive passages (hMSC-Scx cells, P4–P6 and hTSPCs, P5–P7).

### 4.2. Immunocytochemistry

Both cell types were grown on COL1-coated (20 μg/mL) glass slides, and after washing, fixation (4% paraformaldehyde (Merck, Darmstadt, Germany)/PBS) and blocking (2% Bovine Serum Albumin (BSA), anti-CD146, anti-STRO1 (both from Abcam, Cambridge, UK) or anti-Nestin (Proteintech, Manchester, Greater Manchester, UK) antibodies were applied overnight at 4 °C. Next day, corresponding secondary antibodies were added for 1 h. After washing, short counterstaining with 4′,6-diamidino-2-phenylindole (DAPI) (Life technology, Carlsbad, SD, USA) was performed and slides were mounted with fluoroshield (Sigma-Aldrich, Steinheim, Nordrhein-Westfalen, Germany). An automated quantitative image analysis was performed as described in Hsieh et al. 2016 [9] with slight modifications. In brief, using ImageJ (National Institutes of Health, Bethesda, MD, USA), the following algorithm was applied: (1) nine images with a 40× magnification were taken per staining and cell types; (2) areas of all cells were manually designated using the “drawing/selection” tool; (3) the “set measurements” for area, integrated density and mean grey value was selected from the “analyze” menu; (4) lastly, the corrected total fluorescence (CTF) representing the expression of CD146, Nestin and STRO1 was exported and calculated in Excel (Microsoft) as follows: CTF = media of integrated density−media of the selected area x mean fluorescence. Experiments were reproduced three times independently.

### 4.3. PCR

Total RNA from 2D and 3D cell culture of hMSC-Scx cells and hTSPCs was isolated with Qiagen RNeasy Mini kit (Qiagen, Hilden, Germany), and purity (absorbance ratios of 260/280 nm were in the range of 1.8–2.2) was tested spectrophotometrically (NanoDrop, Thermo Scientific Waltham, MA, USA) and then used for quantitative PCR (qPCR). QPCR of tendon-related and other lineage-related genes and collagen cross-linking genes (see Table 1 and Table 2) was performed using RealTime Ready Custom Panel 96–32+ plates (https://configurator.realtimeready.roche.com) according to the manufacturer’s instructions (Roche, Penzberg, Bayern, Germany). Briefly, qPCR reactions were pipetted on ice and each well-contained 10 μL LightCycler 480 probes master mix, 1 μL cDNA and 9 μL PCR grade water. Plates were subsequently sealed and centrifuged down at 2100 rpm for 15 s. Crossing points for each sample were determined by the second derivative maximum method. The house-keeping gene *GAPDH* was used for normalization. Fold change was calculated by the 2^−ΔΔCt^ method, where Δ*C*t = Ct_target_ − Ct_GAPDH_ and ΔΔ*C*t = ΔCt_hMSC-Scx (test)_ − ΔCt_hTSPCs (calibrator)._ For each cell type, qPCR was reproduced three times independently with 2D and 3D cultured cells. In order to evaluate the variability in hTSPCs (the calibrator), the standard deviations were calculated for the mean values of the three samples.

### 4.4. Cell Migration

An automated inverted microscope, AxiovertS100 equipped with AxioCamMRm camera (Carl Zeiss, Jena, Thüringen, Germany) and biochamber (PeCon, Erbach, Baden-Württemberg, Germany), was used. For scratch assay, 1 × 10^4^ cells/cm^2^ were plated on 6-well COL1 (20 μg/mL, Millipore)-coated dishes and were left to form confluent cell layers for 48 h. Prior to imaging, the layers were scratched multiple times using sterile pipette tips. Time-lapse was performed at 4 frames/h for 7 h. The initial area of 9 scratches and then the areas covered by cells at the end of the experiments were measured and used for calculation in %. For analysis of random migration, 1.5 × 10^3^ cells/cm^2^ were plated on 6-well COL1-coated dishes and incubated for 4 h and then imaged at a rate of 4 frames/h for 24 h. The image data were extracted with AxioVisionLE software (Carl Zeiss, Jena, Thüringen, Germany), individual cell tracks (*n* = 320 per cell type) were followed manually, and track lengths were collected with ImageJ software.

### 4.5. Cell Sheets

As described previously [37] upon reaching confluence in 10-cm cell culture dishes, hMSC-Scx cells and hTSPCs were supplemented with high glucose DMEM containing 10% FBS and 50 µg/mL ascorbic acid (A8960, Sigma-Aldrich, Seinheim, Nordrhein-Westfalen, Germany) for 1 week, and then the confluent cell layers were rolled into cell sheets by applying a small roll-up force using a cell scraper. In order to apply static mechanical stretch, the ends of the cell sheets were fixed at 10% elongation in length using sterile metal tips. Cell sheets were left to mature in the same medium for 4 (*n* = 9 per cell type) or 6 weeks (*n* = 9 per cell type). The two time points were selected based on literature [3] and standard chondrogenic pellet culture protocols of 3–4 weeks. The time point of 6 weeks was critical to evaluate if the cell sheets could further mature.

### 4.6. Cell Sheet Histology

The cell sheets were fixed in 4% paraformaldehyde/PBS, cryoprotected with sucrose, embedded in tissue freezing medium (Jung, Leica, Nussloch, Baden-Württemberg, Germany) and cryosectioned at 10 μm (Microm HM500 OM, Fisher, Walldorf, Baden-Württemberg, Germany). Sections were stored at −20 °C until use. Before staining, sections were equilibrated to room temperature and hydrated with PBS (3 × 5 min) and rinsed with deionized water. For Haematoxylin and Eosin (H&E), sections were placed in a ready-to-use Hematoxylin solution (Carl Roth, Karlsruhe, Baden-Württemberg, Germany) for 10 min, rinsed with deionized water for 5 min and washed in tap water for 7 min. Next, sections were immersed in a ready-to-use Eosin solution (Carl Roth, Karlsruhe, Baden-Württemberg, Germany) for 10 min, rinsed with distilled water for 30 s, and mounted. Alexa Flour 546-labelled phalloidin (Invitrogen, Karlsruhe, Baden-Württemberg, Germany) was applied according to the manufacturer’s recommendation, as described previously [6]. DAPI was used for nuclear counterstaining. Cell sheets were imaged with the AxioCamMRm camera on the AxiovertS100 microscope (Carl Zeiss, Jena, Thüringen, Germany). For quantification of the total actin amount, fluorescence signals were quantified using the algorithm described above.

### 4.7. Transmission Electron Microscopy

For sample preparation (three sheets per cell type) and TEM, we used standard procedures as described previously [38]. In brief, specimens were placed into a 4% phosphate-buffered glutaraldehyde solution for fixation. After fixation, initial contrasting was carried outhappened in osmium tetroxide with a concentration of 1%, followed by dehydration in graded concentrations of ethanol. Finally, the specimens were embedded in Araldite. One-micron semi-thin sections were stained with 1% toluidine blue solution. Ultra-thin sections (thickness: 50–70nm) were cut and contrasted with a 2% solution with uranyl acetate and lead according to Reynolds. Examination and imaging of sections were performed with a TEM (Zeiss EM 900, Jena, Freistaat Thüringen Germany) equipped with a digital camera. For analysis of collagen fibril size, 200 collagen fibrils per sheet were measured manually by ImageJ (versionv1.45s, National Institutes of Health, Bethesda, MD, USA) software. 

### 4.8. Statistics

GraphPad Prism 5 software (GraphPad, California, CA, USA) was used for quantitative data and statistical significance analyses. Bar charts show mean values and standard deviations. Statistical testing was performed with a *t*-test, and *p* < 0.05 was considered statistically significant.

## Figures and Tables

**Table 1:** List of tenogenic-related genes expressed in the hMSC-Scx cell line and primary hTSPCs.

Tenogenic-Related Genes	2D Cell Culture		3D Cell Sheets			
			4w		6w	
Cells (S: hMSC-Scx; T: hTSPC)	S	T	S	T	S	T
*Alpha smooth muscle actin (α-SMA)*						
*Cartilage oligomeric matrix protein (COMP)*						
*Collagen, type I, alpha 1 (COL1A1)*						
*Collagen, type III, alpha 1 (COL3A1)*						
*Collagen, type V, alpha 1 (COL5A1)*						
*Collagen, type VI, alpha 1 (COL6A1)*						
*Collagen, type XII, alpha 1 (COL12A1)*						
*Collagen, type XIV, alpha 1 (COL14A1)*						
*Collagen, type XV, alpha 1 (COL15A1)*						
*Early growth response 1 (EGR-1)*						
*Early growth response 2 (EGR-2)*						
*Ephrin type-A receptor 4 (EPHA4)*						
*Eyes absent homolog 1 (EYA1)*						
*Eyes absent homolog 2 (EYA2)*						
*Mohawk homeobox (MKX)*						
*Proteoglycan 4 (PRG4)*						
*Scleraxis homolog A (SCXA)*						
*SIX homeobox1 (SIX1)*						
*SIX homeobox2 (SIX2)*						
*Tenascin C (TNC)*						
*Tenomodulin (Tnmd)*						
*Thrombospondin 2 (THBS2)*						
*Thrombospondin 4 (THBS4)*						
*Transforming growth factor beta 1 (TGF-β1)*						

Green boxes indicate genes highly expressed in the cells, orange boxes indicate genes weakly expressed, and red boxes indicate genes undetectable in the qPCR plates. 4w, 4 weeks; 6w, 6 weeks.

**Table 2:** List of lineage and cross-linking genes expressed in the hMSC-Scx cell line and primary hTSPCs.

Lineage & Cross-Linking Genes		2D Cell Culture		3D Cell Sheets			
				4w		6w	
Cells (S: hMSC-Scx; T: hTSPC)		S	T	S	T	S	T
Adipogenic- related	*Adipocyte Protein 2 (AP2)*						
	*Lipoprotein lipase (LPL)*						
	*Peroxisome proliferator-activated receptor gamma (PPARG)*						
Chondrogenic- related	*Aggrecan (AGCAN)*						
	*Collagen, type II, alpha 1 (COL2A1)*						
	*SRY (sex-determining region Y)-box 9 (SOX9)*						
Embryonic- related	*Fucosyltransferase 4 (FUT4/CD15)*						
	*Nanog homeobox (NANOG)*						
	*Octamer-binding transcription factor 4 (Oct4, Oct3)*						
Muscle- related	*Desmin (DES)*						
	*Myogenic differentiation 1 (MYOD1)*						
	*Myogenin (MYOG)*						
Osteogenic- related	*Integrin binding sialoprotein (IBSP)*						
	*Osterix (OSX)*						
	*Runt-related transcription factor 2 (RUNX2)*						
Collagen cross linker	*Asporin (ASPN)*						
	*Biglycan (BGN)*						
	*Decorin (DCN)*						
	*Fibromodulin (FMOD)*						
	*Fibronectin (FN)*						
	*Lumican (LUM)*						
	*Lysyl hydroxylases (LH)*						
	*Lysyl oxidase (LOX)*						
	*Transglutaminase 2 (TGM2)*						

Green boxes indicate genes highly expressed in the cells, orange boxes indicate genes weakly expressed and red boxes indicate genes undetectable in the qPCR plates. 4w, 4 weeks; 6w, 6 weeks.

## Supplementary Materials

Supplementary materials can be found at http://www.mdpi.com/1422-0067/19/8/2272/s1.
