# Supplementary material for: In Vitro Comparison of 2D-Cell Culture and 3D-Cell Sheets of Scleraxis-Programmed Bone Marrow Derived Mesenchymal Stem Cells to Primary Tendon Stem/Progenitor Cells for Tendon Repair
Source: Int J Mol Sci. 2018 Aug 2;19(8):2272. doi: 10.3390/ijms19082272 (PMC6121892; doi:10.3390/ijms19082272)
Supplement: Supplementary file 1 [file ijms-19-02272-s001.pdf]

Supplementary Materials and Methods

Analysis of cell area

Cells were imaged using with an Axiocam ICc3 camera mounted on an Axiovert 40 CFL microscope (both Carl Zeiss, Goettingen, Germany). HMSC-Scx and hTSPC were cultivated for 24 h and cell areas were measured with the polygonal tool of Image J 1.38 software (NIH open-source program). The average cell area of 32 cells per type was quantified in 2 different passages.

WST-1 and CyQUANT proliferation assays

The WST-1 Proliferation Kit (Roche Penzberg, Germany) was used according to the manufacturer's recommendations. HMSC-Scx and hTSPC (3×10^3 cells/cm^2) were plated in triplicates in 96-well dishes. After 48 h of cultivation, cells were incubated with the WST-1 reagent for 4 h. Optical density was measured using the microtiter-plate reader (Thermo Scientific, Vantaa, Finland) at a wavelength of 420 nm with reference filter at 620 nm. CyQUANT cell proliferation kit was used as recommended by the manufacturer (Invitrogen, Eugene, OR, USA). HMSC-Scx and hTSPC (1.5×10^2 cells/cm^2) were plated in triplicates in 6-well dishes. Cells were frozen 0, 4, 8, 12 and 16 days of cell culture and stored at -80°C. Lastly, cells were lysed with the CyQUANT dye buffer for 5 mins and optical density was measured using the microtiter-plate reader (Thermo Scientific) at 480 nm excitation and 520 nm emission maxima. The WST-1 and CyQUANT experiments were reproduced thrice independently.

Self-renewal analysis

Colony-forming unit (CFU) assay was carried out as follows: 10 cells/cm^2 were plated in 10 cm cell culture dishes. After 14 days, formed colonies were stained with 0.5% crystal violet/methanol staining solution for 10 min and rinsed. Single colonies with 1–8mm diameter were counted and in the case of small cell clusters, a cluster of > 25 cells was considered a colony. Finally, CFU efficiency was determined with the formula CFU [%] = (number of colonies/number of plated cells) x100. CFU assays were performed with hMSC-Scx and hTSPC- in 2 different passages, and each experiment contained triplicate dishes.

Cell differentiation

For adipogenic differentiation, hMSC-Scx and hTSPC were plated in duplicates in 6-well dishes (5 x 10^3 cells/cm^2). Stimulation was started when cells reached full confluency. Cells were stimulated for 21 days using the induction medium composed of DMEM-high glucose medium, 10 % FBS, 1 μM dexamethasone, 0.2 mM indomethacin, 0.1 mg/ml insulin and 1 mM IBMX (all Sigma-Aldrich). Cells were grown for 5 days in induction medium, thereafter for 2 days in maintenance medium and then switched to induction medium again. The extent of adipogenic differentiation was evaluated by AdipoRed assay, which was implemented according to the manufacturer's instructions (Lonza, USA).

For osteogenic differentiation, 3.5 x 10^4 hMSC-Scx or hTSPC/cm^2 were seeded in 6-well dishes and cultivated in osteogenic medium composed of DMEM high glucose, 10 % FBS, 10 mM β-glycerophosphate, 50 μM L-ascorbic-acid-2-phosphate and 100 nM dexamethasone (all Sigma-Aldrich). After 21 days, Alizarin Red staining was performed using the Osteogenic Quantification kit (Millipore, Billerica, USA).

For chondrogenic differentiation, 2.5 x 10^5 hTSPC were seeded in V-bottomed 96-well polypropylene dishes and pellets were formed by centrifugation. Chondrogenic medium composed of DMEM-high glucose medium, 10 μM dexamethason, 1 mM sodiumpyruvat, 0.2 mM L-ascorbic-acid-2-phosphate, 1x ITS (all Sigma-Aldrich), 10 ng/ml TGF-β1 (R&D Systems) and 2 ng/ml BMP2 (R&D Systems) was applied onto the pellets for a culture period of 35 days. Pellets were fixed in 4 % paraformaldehyde and embedded in paraffin. Histological sections (7 μm thick) were stained with toluidine blue and collagen type II or aggrecan anitbodies using standard protocols. Collagen II-positive area was measured with Image J program and expressed in % to pellet area.

Semiquantitative PCR

Total RNA was extracted from hMSC-Scx and hTSPC with RNeasy Mini Kit (Qiagen, Hilden, Germany), and 1 µg RNA was used for cDNA synthesis with AMV kit (Invitrogen) according to the manufacturer' instructions. Semi-quantitative PCR was performed with Taq DNA Polymerase (Invitrogen) in a PTC-200 Thermal Cycler (Bio-Rad Laboratories, Munich, Germany). The cDNA

input was normalized by glyceraldehyde-3-phosphate dehydrogenase (GAPDH) PCR. The PCR products were analyzed on 2% agarose gels. The primer pairs and RT-PCR conditions used in this study are published previously in [11].

Supplementary figure legends

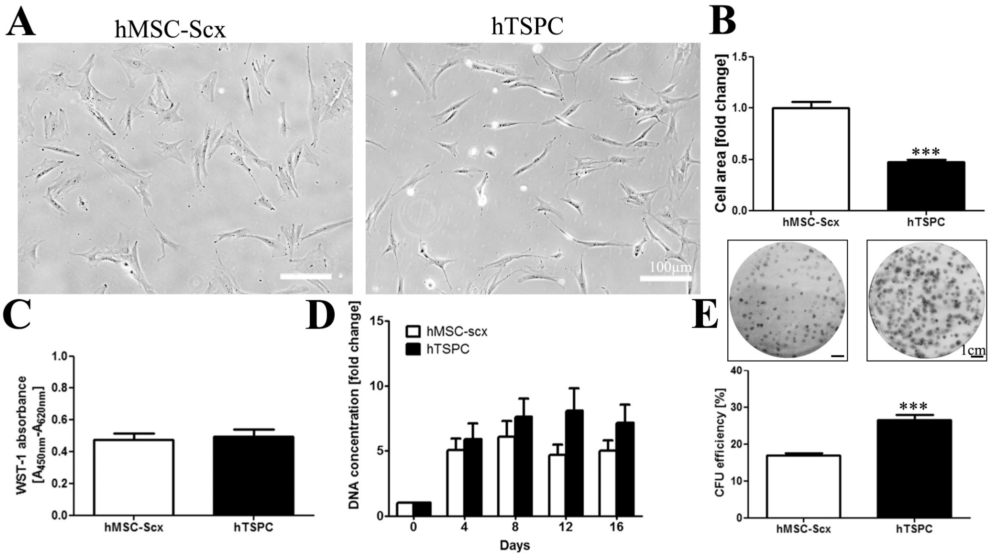

Figure S1. Characterization of hMSC-Scx and hTSPC. **(A)** Representative phase-contrast images of hMSC-Scx and hTSPC and **(B)** quantification of average cell area. **(C and D)** Short- and long term proliferation analyses by WST-1 and DNA-based assays, correspondingly. **(E)** Colony-forming unit (CFU) assay, were formed colonies were visualized by crystal violet staining at day 14. ***p<0.001.

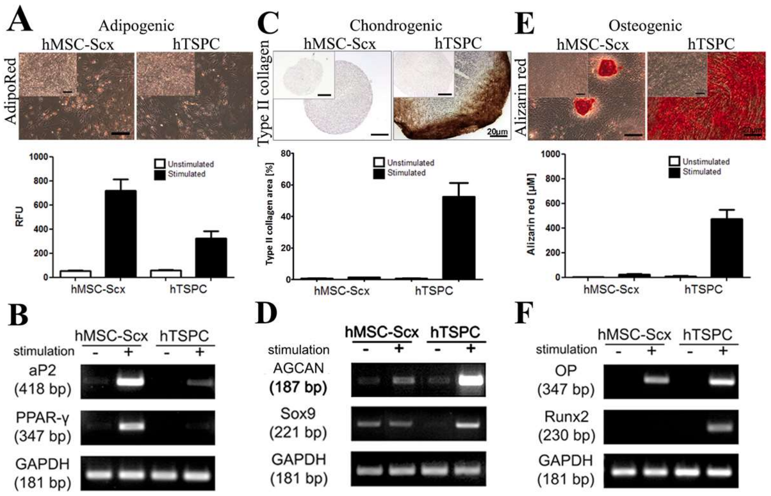

Figure S2. Comparison of a three-lineage differentiation potential of hMSC-Scx and hTSPC. (A) Adipogenic differentiation. At day 21, formed lipid vacuoles were visualized by AdipoRed staining

and quantification of accumulated dye was used to estimate the extent of differentiation. **(B)** Semi-quantitative PCR for the adipogenic markers. **(C)** Chondrogenic differentiation. It was performed for 35 days using pellet cultures and collagen II immunostaining and quantification of positive areas was used to evaluate the degree of differentiation. **(D)** Semi-quantitative of chondrogenic markers. **(E)** Osteogenic differentiation. Alizarin Red staining for calcium deposits was performed at day 21 of stimulation and then used for quantification of osteogenic differentiation. **(F)** Semi-quantitative PCR for osteogenic markers. All differentiation protocols were repeated thrice independently in triplicates for both cell types. In A, C, and E, inserts show unstimulated controls.

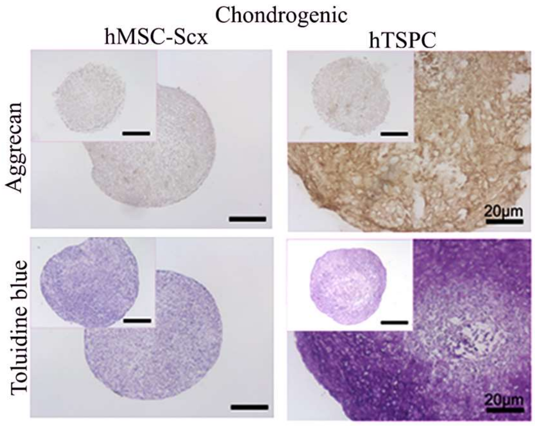

Figure S3. Cartilage-specific staining of hMSC-Scx and hTSPC pellets. Representative images of aggrecan and toluidine blue stainings.
